# Young and Older Adults Differ in Integration of Sensory Cues for Vertical Perception

**DOI:** 10.1155/2020/8284504

**Published:** 2020-07-31

**Authors:** Rima Abdul Razzak, Jeff Bagust, Sharon Docherty

**Affiliations:** ^1^Department of Physiology, College of Medicine and Medical Sciences, Arabian Gulf University, Complex 329, Salmaniyah Road, Manama, Bahrain; ^2^Faculty of Health and Social Sciences, Bournemouth University, Bournemouth BH1 3LT, UK

## Abstract

**Introduction:**

The subjective visual vertical (SVV) measures the perception of a person's spatial orientation relative to gravity. Weighted central integration of vestibular, visual, and proprioceptive inputs is essential for SVV perception. Without any visual references and minimal proprioceptive contribution, the static SVV reflects balance of the otolith organs. Normal aging is associated with bilateral and progressive decline in otolith organ function, but age-dependent effects on SVV are inconclusive. Studies on sensory reweighting for visual vertical and multisensory integration strategies reveal age-dependent differences, but most studies have included elderly participants in comparison to younger adults. The aim of this study was to compare young adults with older adults, an age group younger than the elderly.

**Methods:**

Thirty-three young and 28 older adults (50–65 years old) adjusted a tilted line accurately to their perceived vertical. The rod's final position from true vertical was recorded as tilt error in degrees. For otolithic balance, visual vertical was recorded in the dark without any visual references. The rod and frame task (RFT) with tilted disorienting visual frames was used for creating visuovestibular conflict. We adopted Nyborg's analysis method to derive the rod and frame effect (RFE) and trial-to-trial variability measures. Rod alignment times were also analyzed.

**Results:**

There was no age difference in signed tilts of SVV without visual reference. There was an age effect on RFE and on overall trial-to-trial variability of rod tilt, with older adults displaying larger frame effects and greater variability in rod tilts. Alignment times were longer in the tilted-frame conditions for both groups and in the older adults compared to their younger counterparts. The association between tilt accuracy and tilt precision was significant for older adults only during visuovestibular conflict, revealing an increase in RFE with an increase in tilt variability. Correlation of *σ*_SVV_, which represents vestibular input precision, with RFE yielded exactly the same contribution of *σ*_SVV_ to the variance in RFE for both age groups.

**Conclusions:**

Older adults have balanced otolithic input in an upright position. Increased reliance on visual cues may begin at ages younger than what is considered elderly. Increased alignment times for older adults may create a broader time window for integration of relevant and irrelevant sensory information, thus enhancing their multisensory integration. In parallel with the elderly, older adults may differ from young adults in their integration of sensory cues for visual vertical perception.

## 1. Introduction

The subjective visual vertical (SVV) is a psychophysical measure of the angle between perceptual vertical and true (gravitational) vertical [1]. SVV clinical test evaluates a person's ability to align accurately a linear marker (i.e., a rod) to gravitational vertical (0°). The ability to judge whether the linear marker is aligned with the real vertical depends on the integrity of visual [2–4], vestibular otolithic [4–8], and somesthetic information [9–11]. This information codes the static gravitational orientation and cephalic linear acceleration movements, with consequent maintenance of posture and balance [4, 12]. The static SVV is tested in complete darkness, in order to exclude visual references, and in an upright sitting position, so that proprioceptive inputs contribute only minimally. Assessed in such conditions, the static SVV reflects tonic otolithic input differences between the two ears [13, 14].

It is well known that aging brings about significant changes in all sensory systems and a variety of cognitive functions. With respect to sensory modalities important for SVV, visual acuity and somatosensory along with vestibular function tend to decrease with age [15, 16]. Age-related vestibular changes include hair cell loss, neuronal loss beginning around the age of 55 years [17, 18], degeneration of the vestibular ganglion and nerve [19], reduced blood flow to the inner ear, and increased severity of idiopathic bilateral vestibular loss [17]. Earlier vestibular changes include a decrease in size and number of neurons in the vestibular nucleus at a rate of 3% each decade beginning around the age of 40 years [20]. A recent study on mice has reported that the loss of extrastriolar calyceal synapses has a key role in age-dependent vestibular dysfunction [21]. The functional consequence of decline in the function of the otolith organs includes age-dependent reduction in afferent signals to the integrating centers for SVV within the central nervous system, and consequently reduced sensitivity to gravity and linear acceleration [22, 23]. Despite this age-dependent deterioration, reports on age-dependent modulation of SVV are inconsistent [24–28].

According to Bayesian theory, multisensory integration occurs in a weighted fashion based on the reliability of the component sensory signals [29–31], and the outcome of this integration process is a single percept. Normally, in a situation of sensory conflict, the central nervous system first identifies the discrepancy and reduces the weighting of inaccurate or noisy input while increasing the weight of input from the sensory systems deemed to provide more reliable (less noisy) information [32, 33]. In bilateral vestibulopathy, patients reweight the remaining extravestibular sensory information relying more on visual and other nonvestibular inputs compared with healthy controls in the perception of spatial orientation [34]. As for the elderly, Alberts et al. [35] have shown that, due to progressive bilateral vestibular deterioration, they too compensate for the sensory deterioration by reweighting sensory inputs. By using Bayesian inference, the authors deduced that, for perception of vertical with visual contextual information, there is an age-dependent reweighting of sensory information and shift in its sensory weights favoring visual contextual information and downweighting of unreliable and noisy vestibular signals.

In addition to age-related anatomical and functional sensory changes, another important aspect of aging is changes in multisensory integration strategies. Despite the age-associated sensory loss, deterioration of sensory processes, and age-related cognitive slowing [36, 37], the elderly exhibit greater integration of multisensory stimuli than younger adults. The few proposed potential mechanisms of enhanced multisensory integration in the elderly include the following: (1) alterations in the temporal parameters of integration, with the elderly having a broader time window of integration as a consequence of increased response times [38, 39]; (2) deficits in top-down attentional control and modulation of sensory processing that allows more distraction by stimuli within and across sensory modalities [40–42]; (3) principle of inverse effectiveness, which represents the fact that reduced sensitivity or acuity (effectiveness) of individual sensory stimuli increases the magnitude of multisensory enhancements, as a compensatory strategy to counteract the detrimental consequences of unisensory deterioration [39, 43]; (4) elevated baseline levels of background sensory processing leading to processing of stimulus regardless of their relevance, inadequate filtering of sensory noise, and eventually greater distraction when incoming sensory streams contain irrelevant or conflicting information [40, 44].

There is no consensus on the age at which people are considered elderly, but in some countries, the elderly are defined as having a chronological age of 65 years or older [45]. Most studies on aging of SVV have considered participant age in a continuum or compared SVV mainly between young adults and age groups above 60 years [26, 27]. Even though sensory, specifically vestibular, impairments begin in the fifth decade of life [20], not many studies compared verticality perception between young adult and advanced age groups, older than 50 years but not categorized as elderly. Additionally, while the age effects on visual dependence are progressive in nature [46], it is uncertain whether age-dependent differences in sensory reweighting between young and older adults categorized as nonelderly are as profound as those between young adults and the elderly. In the present study, we investigated verticality perception and visual dependence in young adults and in a group of adults with an age range of 50 to 65 years. We refer to this group as “older adult.”

The rod and frame test (RFT) can characterize specifically the weighting of visual and vestibular cues in the estimate of verticality when there is incongruency between visual and vestibular inputs, such as introducing on the RFT an oriented square frame around the rod. The surrounding orientation perturbation serves as an inaccurate and distracting visual cue for SVV perception. Most individuals deviate from the true vertical toward the tilted frame, an effect known as the rod and frame effect (RFE) [47]. We hypothesize that, in older adults, the potentially reduced vestibular information may influence the perception of vertical and reweighting of associated sensory inputs on the RFT, just as in the elderly. As older adults may suffer from progressive bilateral vestibulopathy, we expect that they would reduce the weighting of unreliable vestibular information and increase the weight of visual cues for identification of vertical.

We will also attempt to relate our findings to age-related changes in multisensory integration. Assuming that older adults may be more prone to enhanced multisensory integration, just like the elderly [40], we expect inferior performance due to a visual distractor on the RFT compared to young adults. Accordingly, the aim of the present study is to compare the following between young and older adults: (1) static SVV tilts in the frontal plane and levels of visual dependence on the RFT; (2) trial-to-trial variability as a measure of SVV precision, which depends mostly on otolith input in the upright position [48]; (3) response times of rod adjustments despite the lack of time restrictions for the task. Findings may help determine whether there are age-dependent differences in sensory reweighting of vestibular and visual cues and multisensory integration strategies at a relatively younger age groups compared with the elderly.

## 2. Materials and Methods

All experiments were conducted in accordance with the Declaration of Helsinki. This study was approved by the Research and Ethics Committee (REC) in the College of Medicine and Medical Sciences (CMMS) at the Arabian Gulf University (AGU). All participants provided written informed consent to take part in this study.

### 2.1. Participants

Only one participant was above our age limit of 65 years (69 years) and was consequently excluded from the study. Measurements of SVV were made in 33 young healthy male adults (mean age = 21.2 ± 2.70 years) who were medical students at AGU and 28 older healthy participants (20 males and 8 females) with a minimum age of 50 years (mean age: 57.02 ± 5.63 years; range: 50–69 years). The older participants were members of the academic or administrative staff at CMMS, AGU. Of the eight females in the older adult group, four were older than 60 years of age. Females were excluded from the young adult group due to menstrual cycle effects on visual vertical perception [49].

All participants were right-handed based on self-report. Participants were excluded if they had a history of any previous sensation of dizziness, vertigo, migraine, and neurologic or metabolic disorder. All participants had normal or corrected-to-normal vision.

### 2.2. Measurement of SVV: The Computerized Rod and Frame Test (CRFT)

**Figure 1:**
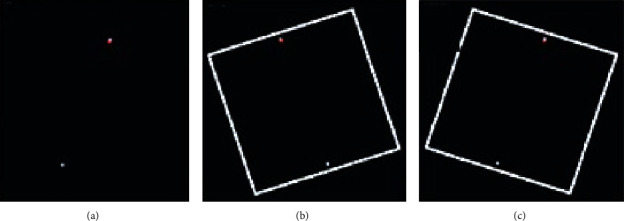
Presentation on the computer screen of the three frame conditions of the computerized rod and frame test (CRFT). (a) No-frame (*q, r* combinations). (b) Frame^−18°^(*u*, *v* combinations). (c) Frame^+18°^ (*w, x* combinations).

We have utilized a computerized version of the rod and frame test (CRFT) to assess verticality perception. This test is a modified version of the RFT [50, 51], in which a virtual line marked by two white dots at its ends was used instead of a continuous line to reduce clues to verticality, which might be provided by the stepped appearance of a displayed solid line. There is no difference between the rod and dots presentations in the measurement of SVV [51]. The test was performed while sitting in a comfortable position with no head restraint; however, participants were instructed to keep their trunks and heads fixed and maintain their feet in a flat position. The virtual rod was viewed in a two-dimensional (2D) display through head mounted video eyeglasses (VUSIX iWEAR, VR920 Video Eyewear) (Figure 1(a)) restricting the field of vision and providing an image that spanned an angle of 30 × 23 degrees of the visual field (the equivalent of viewing a 1.42 m screen from a distance of 2 m). Where necessary, the video eyeglasses were used over spectacles.

Participants rotated the dots around their virtual center in 0.5° increments in either clockwise (CW) or counterclockwise (CCW) directions using the mouse buttons until the “rod” was considered vertical. The space bar of the computer keyboard was then pressed to record the rod alignment relative to vertical and move the program to the next presentation. Recording of rod alignment tilt on the RFT test was conducted on 14 presentations in total. The first two presentations were for demonstration of the test and used to confirm that the participant understood the task. These measurements were not included in the analysis.

For the remaining 12 presentations, visual vertical was measured in three visual contexts (Figure 1) with four trials for each visual context: no visual reference\frame (dark SVV); the frame tilted counterclockwise (−18°, Frame^−18^) and tilted clockwise (+18°, Frame^+18^) with respect to the vertical. To eliminate possible tilt and learning effects, the order of display presentations was randomly selected by the computer at the beginning of each trial from a bank of four sequences for each frame condition. Participants were informed of the importance of spatial accuracy, and there was no time restriction for completing the task.

### 2.3. CRFT Analysis

The angular deviation of the rod's final position from true vertical was recorded as error in degrees. According to convention, clockwise (CW) tilts of the rod by the participants were denoted by a positive value, whereas counterclockwise (CCW) tilts were considered negative. Two different SVV analyses were performed: central tendency of signed SVV tilt to reflect accuracy of SVV alignment and intraindividual variability of SVV tilt across the four trials for precision of SVV alignment.

#### 2.3.1. Signed Mean Tilt

The objective of this analysis was to determine otolithic tonus balance, since SVV tilts in the dark with minimal proprioceptive contribution are known to be a sensitive sign of otolithic tone imbalance [52, 53]. For that, we utilized the signed values of tilts during conditions without any visual reference (Dark SVV). This gives a measure of the individual's internal representation of SVV.

#### 2.3.2. Nyborg's Analyses on the RFT

We also analyzed the data according to Nyborg's method [54] for the rod and frame test (RFT), in which different variables were derived from the raw signed tilt values during tilted-frame conditions. Due to the three different frame conditions and two starting positions for rod (counterclockwise −20° and clockwise 20°), there are six different combinations of frame and rod conditions used in the CRFT (Table 1).

*(1) Constant Error, μ*. This is expressed as the mean of all signed alignment tilts during the eight trials of tilted frame (4 CW and 4 CCW). It represents the central tendency to adjust a rod consistently to one side of vertical (0°).

*(2) Frame Effect, φ*. In addition to reporting mean signed deviation tilt during tilted-frame condition, we analyzed these data according to Nyborg's recommended method for evaluating visual dependence and response variability on the RFT. Nyborg's frame effect variable is an important measure of visual dependence. It accounts for the tilt of the frame (CCW or CW) and rod starting position (CCW or CW). Most importantly, this variable can evaluate reweighting of visual and vestibular references as it represents the attraction of a perturbing visual field on the subjective vertical. Higher values indicate that a visual strategy is mainly used to estimate verticality while low values indicate that verticality is mainly estimated using a proprioceptive or vestibular strategy.

Nyborg described the frame effect as follows: for each of the two conditions of frame tilt, the effects of counterclockwise and clockwise rod starting positions are counterbalanced (Table 1). Therefore, the frame effect, *φ,* can be found as the mean of the four observations, in the “frame tilted” condition. Since the subject's constant error, *μ*, contributes to all observations, it is subtracted from this mean. By definition, the two values of *φ*, based on the two conditions of frame tilt, will be exactly symmetrical. Therefore, we only needed to analyze one frame tilt condition (clockwise; +18°) in this study:

(1) $$\begin{array}{c} \varphi =\;\frac{\;w_{1}+w_{2}+x_{1}+x_{2}}{4}\;-\;µ\;. \end{array}$$

*(3) Intraindividual Variability Measure*. An analysis was done to detect intraindividual variability about verticality perception. This measure, calculated as the standard deviation of the repetitive tilts, reﬂects the precision of the tilts in the roll plane. An increased intraindividual variability does not necessarily indicate an otolithic tone imbalance but is considered to be a decreased effectiveness of the otolithic organs [48, 55]. It is equivalent to Nyborg's “response consistency,” an estimate of the variability of the participant's signed tilts in four trials in different frame conditions and both starting positions of the rod. A large value of variability measure indicates that the participant is not responding consistently, or in other words not with precision.

We calculated the variability measure “*σ*_SVV_” as standard deviation (SD) around the mean signed tilt, for the two combinations with no visual reference (no frame) using

(2) $$\begin{array}{c} \sigma_{\text{SVV}}=\text{SQRT}\left(\frac{\left({\left(q_{1}-q_{2}\right)}^{2}/2\right)+\left({\left(r_{1}-r_{2}\right)}^{2}/2\right)}{2}\right). \end{array}$$

For the four combinations representing the tilted-frame conditions (Frame^−18°^ and Frame^+18°^), the equation for the variability measure “*σ*” is given by

(3) $$\begin{array}{c} \sigma =\text{SQRT}\left(\frac{\left({\left(u_{1}-u_{2}\right)}^{2}/2\right)+\left({\left(v_{1}-v_{2}\right)}^{2}/2\right)+\left({\left(w_{1}-w_{2}\right)}^{2}/2\right)+\left({\left(x_{1}-x_{2}\right)}^{2}/2\right)}{4}\right), \end{array}$$

where *u* and *v* represent Frame^−18°^ combinations and *w and x* represent Frame^+18°^ combinations (Figure 1 and Table 1). The subscripts 1 and 2 represent the trial number for the same rod starting position.

#### 2.3.3. Alignment (Response) Time

The mean time taken to complete the alignment for each of the three frames (no frame, Frame^−18°^, and Frame^+18°^) was calculated, and a combined frame tilted (CFT) time was calculated by averaging the Frame^−18°^ and Frame^+18°^ times, since there was no significant difference between the two directions of frame tilt for both groups (young adults: paired *t* (31) = 1.15, *P*=0.26; older adults: paired *t* (26) = 1.150, *P*=0.28). To account for generalized cognitive slowing, mean response times were evaluated after log transformation [56–58].

### 2.4. Statistical Analysis

Data were analyzed using the IBM SPSS Statistics 25 software. Signed values of alignment tilts were used. Data were tested for normality using the Kolmogorov–Smirnoff method. For each of response variability and log response time, a 2 × 2 repeated measures ANOVA with age as the between-subjects factor (young adults; older adults) and frame condition as the within-subjects factor (no frame; tilted frame) was used. The partial eta squared (*η*^2^) was used to determine the effect size. For comparing alignment tilt measures between the two age groups, we used Student's *t*-test, since RFE measure, representing the visual dependence, was derived, rather than being raw data, as is the case for SVV in the no-frame condition. Correlation between variability and rod tilt accuracy measures was carried out with Pearson correlation analysis. Data were reported as mean ±SD, and level of significance was set at *P* < 0.05. Any data values exceeding mean ±3SD were considered outliers and were not included in the analyses.

## 3. Results

**Figure 2:**
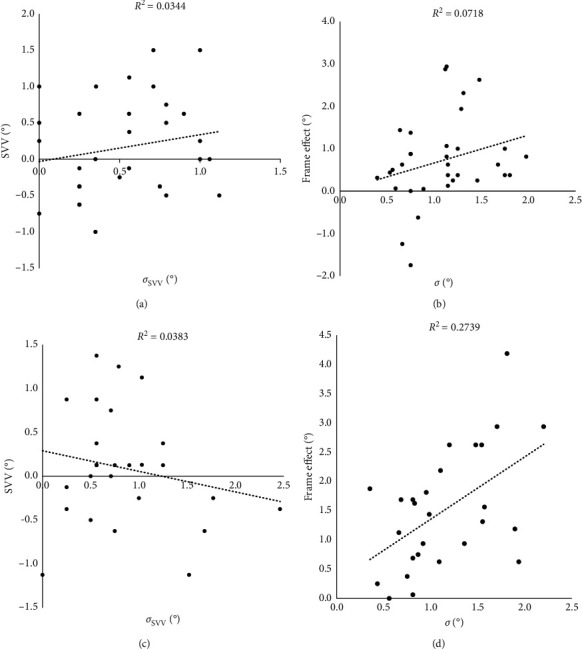
Associations between intraindividual tilt variability and tilt magnitude or frame effect in young adults (a, b) and older adults (c, d). *σ*_SVV_ and *σ* represent variability in the no-frame and oriented-frame conditions, respectively.

All SVV measures passed normality for both age groups. In the young adult group, most tilts were within the normal range for the associated frame condition (<2° for no-frame and <4° for tilted-frame conditions) (Figure 2), and there were no outlier data for all frame conditions. In the older adult group, one participant had mean tilt of −7.88° in the Frame^−18°^ condition; since this value was beyond mean −3SD of −6.67°, all tilt values for this participant were discarded. Another older adult participant had mean tilt of 6.63° in the Frame^+18°^ condition, but this value did not exceed mean +3SD of 5.79°. Consequently, for the older adult group, the remaining number of data points included in the analysis was *n* = 27.

Table 2 displays the means and the range of signed tilts in both age groups and all frame conditions. There was no difference in signed tilts of SVV between the two age groups in the no-frame condition (*t* (57) = 0.42, *P*=0.67). The difference in the constant error value between the two age groups was not significant either (*t* (57) = 1.21, *P*=0.27). Older adults had a significantly larger frame effect by 0.80° than young adults (*t* (57) = 2.99, *P*=0.004), suggesting they were more visual field dependent than the young adult group.

Table 2 displays the means and the range of alignment times and log-transformed time in both age groups. CFT data for the older adults did not pass normality testing. The range of mean alignment times was wider for the older adult group for both the no-frame and CFT measures. The 2 × 2 repeated measures ANOVA on log time yielded significant main effect of age (*F* (1, 57) = 5.67, *P*=0.021, *η*^2^ = 0.090) and of frame condition (*F* (1, 57) = 62.76, *P* < 0.0001, *η*^2^ = 0.524), but no interaction between age and frame condition (*F* (1, 57) = 0.12, *P*=0.723). Irrespective of frame condition, older adults had greater alignment times than their younger counterparts, and irrespective of age, the alignment time was greater in the tilted-frame conditions compared with the no-frame condition.

Intraindividual tilt variability values are also presented in Table 2. The 2 × 2 repeated measures ANOVA of variance on variability values revealed that the main effect of age yielded an *F* ratio of *F* (1, 57) = 4.94, *P*=0.030, *η*^2^ = 0.082, while that of frame condition yielded *F* (1, 57) = 36.43, *P* < 0.0001, *η*^2^ = 0.398. The interaction effect between age and frame condition was nonsignificant (*F* (1, 57) = 2.14, *P*=0.149). There was significantly greater overall variability for the older adults compared with the younger adults and in the tilted-frame condition compared with the no-frame condition.

Correlation analysis (Figure 2) between intraindividual tilt variability and rod tilt (*σ*_SVV_ and SVV) or frame effect (*σ* and RFE) revealed no association between trial-to-trial tilt variability and magnitude of tilt when there was no frame (young adults: *r* = 0.187, *P*=0.298; older adults: *r* = −0.196, *P*=0.328). For oriented-frame conditions, there was a significant increase in frame effect with increasing tilt variability only for the older adult group (young adults: *r* = 0.268, *P*=0.131; older adults: *r* = 0.521, *P*=0.005). Interestingly, correlation between *σ*_SVV_ and RFE yielded an exact level and direction of association in both age groups (young adult: *r* = −0.154, *P*=0.40; older adults: *r* = −0.153, *P*=0.45).

## 4. Discussion

Age-related vestibular changes start from an age well below 50 years [59], and vestibular impairments begin in the fifth decade of life [20]. Chronic degenerative hair cell loss of the otoliths organs and semicircular canals is a main age-related cause of vestibular dysfunction [17]. We investigated whether SVV, a spatial orientation task, and visual dependence on the rod and frame test (RFT) may differ between a group of young and another group of older adults, less than 65 years of age, in order to identify whether performances in this age group are in parallel with those of the elderly. We discussed our results in terms of sensory weighting and multisensory integration involved in perception of vertical. In light of contribution of the somatosensory inputs to verticality perception and that it too may become less reliable with age [15], static SVV was assessed with the head and body in the upright position, with minimal proprioceptive cues from the feet and no external visual cues.

Mean SVV tilt values in our study were within the normal range, as it is well established that normal values of static SVV in healthy people vary from −2.0 to +2.0 degrees [1, 47]. Despite well-documented findings regarding the aging effect on the vestibular system [17–23], we found no effect of age on SVV. Such results support previous studies that showed that spatial accuracy for SVV is not impaired nor intensified with age [26–28]. However, our results are in disagreement with the results reported by Baccini et al. [25], who reported that static SVV measurements were age-dependent and that older participants had more difficulty in judging the absolute vertical, resulting in larger deviations from the true vertical.

Our results are in accordance with those reported by Verhagen et al. [60], who showed that bilateral vestibular deterioration does not result in significant impairments of SVV. A possible reason for preserved perception of vertical in the older adult group lies in how aging affects the vestibular system. Unlike acute vestibular disorders that result in impaired SVV, vestibular changes associated with aging are subtle, accumulating over years of life. Such progressive loss of vestibular function with aging may not affect SVV prominently for the following reason. Aging brings about gradual and bilateral vestibular disorders [61], but if vestibular effects were unequal bilaterally, then the balance of the utricular tone could possibly be disrupted. However, regular long-term central compensatory mechanisms will take effect to recalibrate the utricular inputs to reduce any asymmetry of responses from both sides [62, 63]. Alternatively, if the aging processes affected both sides symmetrically, there may be a relatively equal reduction of utricular input to the vestibular nuclei. With symmetrical bilateral utricular weakness, there may be no detectable difference in SVV [64].

In the current study, analysis of perception of visual vertical with the Nyborg's method clearly distinguished between young and older adults. Introducing a disorienting visual frame resulted in larger alignment tilts in both age groups in our current study, a phenomenon known as the rod and frame effect (RFE) [47]. However, there was a significantly larger frame effect in older adults, indicating that older adult participants were more reliant on visual surround information for establishing verticality compared to their younger counterparts. The difference in RFE was 0.80°, which is greater than the resolution of the recording system (0.50°), indicating a functional difference. This suggests a greater impact of the visual surround manipulations on older adult perception of vertical and indicates differences in weighing sensory information. Such findings are in accordance with those found in the elderly and confirm the age-dependent increase in visual dependence reported in the literature [24, 32, 35, 65–67].

Our findings of greater intraindividual variability in older adults irrespective of frame condition are to some extent in accordance with those reported by Alberts et al. [35]. However, in that study, intraindividual variability for vertical perception was compared separately for no-frame conditions and during visuovestibular conflict, and larger variability with increasing age was found only during visuovestibular conflict.

Intraindividual response variability has often been conceived as noise, reducing the signal-to-noise ratio [68, 69]. It has been suggested that, with age, there is an increasing additive noise of the vestibular system attributed to a reduction in vestibular function [70]. Such age-related increased noise of the vestibular system may explain that when reweighting visual and vestibular inputs for estimation of gravity direction, older adults, just like the elderly, may rely more on visual contextual cues. For the older adults in this study, we proposed age-related bilateral vestibular deterioration and reduced sensitivity of the otolithic organs. Such a defect will render the vestibular signal unreliable and noisy. The greater trial-to trial variability in tilt for the older adults in this study may represent increased noise levels compared with younger adults. One cannot ignore that, during the trials with a tilted surrounding frame, the visual frame information is also unreliable and can add to noise. However, the presence of a greater frame effect for the older adults suggests that they opted to rely more on visual cues, even though they were unreliable. The present findings of larger frame effects (greater visual dependence) and increased response variability in older adults are consistent with previous aging [24, 67] and clinical [61, 69] reports on increased visual reliance when vestibular information becomes less reliable.

When comparing the overall intraindividual variability irrespective of age, there was higher tilt variability during the oriented visual context in comparison to absence of frame. Such results are similar to those reported by Alberts et al. [70], who demonstrated that intraindividual variability of response tilts was larger in oriented-frame conditions; however, their comparison was with an upright frame. This could be a consequence of the higher cognitive demand during the more difficult presentations with the tilted surrounding frame. No assessment was made of the participant's subjective impression of difficulty, but participants frequently commented that they found the tilted-frame trials more difficult.

Our results on correlation between variability and magnitude of tilt deviations or frame effect highlight a new aspect to the age-related effects on visual-vestibular interactions for perception of vertical. They also offer validation to the age-related differences in associations between sensory noise and accuracy of verticality perception. In the present study, SVV variability and SVV tilts for young adults were not correlated, neither were frame effect and variability during tilted frame condition, suggesting that the precision and accuracy of sensory contributors to perception of vertical and spatial orientation are dissociable in young adults. This pattern is in agreement with other studies that suggested that SVV alignment accuracy and precision, depending mostly on otolith afferent input, are not linked [48, 71]. For older adults, however, such associations were frame dependent. With no visual cues, precision of alignment tilts and their mean accuracy were not correlated. Yet, during visuovestibular conflict, there was a significant correlation between the two measures, with intraindividual variability contributing to 27% of the variance in frame effect (RFE). As intraindividual variability increased, or precision decreased, the frame effect increased, or accuracy decreased.

Correlation of *σ*_SVV_, which represents vestibular input precision, with RFE yielded the same contribution of *σ*_SVV_ to the variance in rod and frame effect (RFE) for both age groups. One would expect a higher contribution of vestibular noise to the decrement of accuracy in older adults, since *σ*_SVV_ was greater for older adults than the younger adults by a factor of 1.58 (older adult/young adult: 0.84/0.53). A possible interpretation is that older adults might have downweighted the vestibular component to levels similar to young adults and that the majority of variability or noise arose from the tilted visual frame. This would indicate an increased weight of the visual input and would be in line with previous findings by Alberts et al. [35] of an age-dependent shift in sensory weights favoring visual contextual information and downweighting of unreliable and noisy vestibular signals.

The difference in rod alignment times between younger and older adults in the current study may provide another explanation in terms of multisensory integration strategies. Different strategies of central processing of multisensory information for spatial orientation may be a reason for the increased visual dependence in the older adult participants in this study. This is expected as changes of perceptual and cognitive processes and underlying structural and functional brain changes during healthy aging can alter multisensory integration strategies throughout the lifespan. Comparison of alignment time revealed that older adults took longer to align the rod to their perceived vertical. As mentioned earlier, mean alignment times were evaluated after log transformation which can help to equate response times from young and older adults and correct for differences related to general cognitive slowing. The longer response times for the older adults could provide a broader time window to integrate more information and enhance the use of multisensory integration. On the negative side, this could grant them a longer time period to direct their attention to the distracting visual frame in addition to the rod during tilted-frame conditions. This would be nonbeneficial if older adults have deficits in selective attention, just as in the elderly. Deficits in attentional control in the elderly have been proposed as a reason for the increased amount of multisensory information being integrated, as they fail to focus on the attended stimulus but rather integrate all the information available [39, 72]. This is especially pertinent when target information is physically integrated with distracting information. [73].

For both age groups, there were longer alignment times for presentations of visuovestibular conflict than presentations without any visual cues. Although there was no difference in the distance when the rod had to be rotated in the tilted- and no-tilt-frame conditions, the increased time taken when the frame was tilted appeared to correspond to an impression of increased level of difficulty.

Finally, increased sensory noise at baseline, just like in the elderly, could explain differences in the ability to ignore the distracting tilted frame between the younger adults and older adults in the current study and offer an explanation for the differences between younger and older adults in the weighting of the sensory information on RFT. It has been proposed that increased noise at baseline in the elderly leads to more sensory noise and could hinder the elderly judging if the information is irrelevant or unreliable [40]. Imaging studies have shown that when the elderly engaged in selective attention, there was higher activation of multisensory areas than young adults, meaning they could not successfully ignore nonrelevant information [74, 75]. It is possible that older adults in this study failed to either detect that the information from the tilted frame is unreliable and/or to inhibit the use of this unreliable information.

We have previously reported gender differences in visual vertical perception depending on menstrual cycle phase in females [49]. This is the reason why young females were excluded from the study. The inclusion of females in the older adult group produces a gender imbalance, which may be a limitation of the current study. However, it is unlikely that the results are confounded by sex differences in the older adult group, since only 30% (8/27) of the participants in that group were females between the age of 50 and 64 years, 50% of which were above the age of 60, well above the average age of menopause [76].

## 5. Limitations and Conclusion

One limitation of this study is that the head position was not controlled during the experiment; however, even when the head was not restrained, the effect of vestibular cues was minimized by instructing the subjects to keep their head upright and as still as possible. Additionally, our experimental procedure did not include modulation of verticality perception during head tilts, which would affect vestibular noise and vestibular variance, thereby further altering its weight when combined with a visual cue.

Despite these limitations, difference in sensory weighting mechanisms and multisensory integrating strategies can offer potential explanations for the difference in performance between younger and older adults on the RFT, just like the elderly. The frame effect and variability measures in the head upright position distinguished between young and older adults in their level of visual dependence, indicating that increased reliance on visual cues may begin at ages younger than what is considered elderly. Results point to vulnerability of older adults to multisensory processing changes just like the elderly and to some aspects of enhanced multisensory integration in the older adults compared to their younger counterparts. In parallel with the elderly, older adults may differ from young adults in their integration of sensory cues for visual vertical perception.

## Figures and Tables

**Table 1:** The six different combinations of frame and rod conditions.

Frame condition	Rod starting position	
	Counterclockwise (−20°)	Clockwise (+20°)
No frame	*q*_1_, *q*_2_	*r*_1_, *r*_2_
Frame^−18°^	*u*_1_, *u*_2_	*v*_1_, *v*_2_
Frame^+18°^	*w*_1_, *w*_2_	*x*_1_, *x*_2_

**Table 2:** Means and intraindividual variability of alignment signed tilts on the CRFT for young and older adults.

		No frame	Frame^−18°^		Frame^+18°^

*Alignment tilt*°	Young adult	0.17 (0.66) (−1.00 – 1.50)	−0.83 (1.31) (−4.5 – 1.50)		0.61 (1.53) (−2.00 – 4.25)
	Older adult	0.09 (0.65) (−1.13 – 1.38)	−1.55 (1.12)^*∗*^ (−3.63 – 0.63)		1.47 (1.46)^*∗*^ (−0.50 – 6.63)

		No frame	CFT		

*Alignment time* (*s*)	Young adult	8.70 (2.61)	11.69 (4.55) (5.62 – 24.50)		
		(5.19 – 15.04)			
	Older adult	10.68 (3.43) (6.15 – 20.62)	14.39 (5.92) (8.34 – 31.90)		
*Log* (*time*)	Young adult	2.19 (0.29) (1.65 – 2.71)	2.42 (0.39) (1.73 – 3.28)		
	Older adult	2.33 (0.29) (1.82 – 3.03)	2.60 (0.36) (2.12 – 3.50)		

		Constant error	Frame effect	*σ*_SVV_	*σ*

*Nyborg's analyses*	Young adult	−0.11 (0.98) (−1.63 – 2.31)	0.72 (1.02) (−1.75 – 2.94)	0.53 (0.33) (0.00 – 1.12)	1.08 (0.42) (0.40 – 1.98)
	Older adult	0.18 (0.99) (−1.31 – 2.44)	1.51 (1.02) (0.00 – 4.19)	0.84 (0.54) (0.00 – 2.46)	1.14 (0.52) (0.35 – 2.20)

Values represent mean (SD). The range is also included. *σ*_**SVV**_ and *σ* represent trial-to-trial variability in the no-frame and oriented-frame conditions, respectively. CFT represents the average of time of rod alignment for Frame^−18°^ and Frame^+18°^ presentations. Log time: log-transformed mean alignment time with base “*e*.” All parameters in Nyborg's analyses are in degrees. Young adult: *n* = 33; older adult: *n* = 27. ^*∗*^Significant difference at *P* < 0.05.

## Data Availability

The data used to support the findings of the study are available from the corresponding author upon request.
